# Blasting profile evaluation of sand-mud interbedded surrounding rock during the large-span tunnel construction

**DOI:** 10.1038/s41598-024-62921-3

**Published:** 2024-05-30

**Authors:** LongHao Ma, Fei Lin, Yanqiang Du, Song Ren, NengZeng Long, Ping Zhang

**Affiliations:** 1 School of Civil Engineering, Luoyang Institute of Science and Technology, Luoyang, 471023 Henan China; 2 School of Resources and Safety Engineering, Chongqing University, Chongqing, 400030 Chongqing China; 3 China Coal Technology Engineering Group Huaibei Blasting Technology Research Institute Limited Company, Huaibei, Anhui 235099 People’s Republic of China; 4 State Key Laboratory for the Coal Mine Disaster Dynamics and Controls, Chongqing University, Chongqing, 400044 China

**Keywords:** Layered surrounding rock, Tunnel construction, Blasting optimization, Numerical simulation, Blasting effect evaluation, Civil engineering, Petrology

## Abstract

The sand-mud interbedded surrounding rock contains discontinuities, such as horizontal bedding, joints, weak planes and weak interlayers. Drilling and blasting construction in this kind of surrounding rock is very likely to cause very serious over-/under-excavation phenomenon and excessive damage to surrounding rock, and the contour flatness after smooth blasting of the tunnel is also difficult to be guaranteed, which increases subsequent construction procedures and reduces production efficiency. In order to effectively evaluate the smooth blasting effect of the sand-mud interbedded surrounding rock tunnel, taking a tunnel project in southwest China as the research background, the blasting numerical simulation of the sand-mud interbedded surrounding rock tunnel was carried out using the dynamic analysis program, and the corresponding blasting optimization scheme was obtained. Subsequently, based on fuzzy mathematical theory, the evaluation system of blasting effect of sand-mud interbedded tunnel was established by combining the evaluation criteria of tunnel smooth blasting quality. Immediately afterwards, the weights of each influencing factor index were determined, and the blasting shaping effect of the original blasting scheme and the optimized blasting scheme was evaluated. Finally, the results have shown that the optimized tunnel blasting profile effect was better than the original blasting scheme. The corresponding research results have certain guiding significance for similar tunnel blasting effect evaluation and blasting parameter design.

## Introduction

In recent years, various countries have invested heavily in infrastructure development such as highways and railways, which has greatly contributed to inter-regional economic growth and cultural exchange. As a key part of the construction of the transportation system, tunnels play an extremely important role in ensuring national strategic security, material transfer, basic livelihood, transportation interconnection, etc. At present, tunnel boring generally uses blasting, shield and TBM construction methods, but for some special geological conditions, such as large buried depth, layered surrounding rock, weak interlayer, water-rich zone, karst development, etc.^1^, there are many difficulties in mechanical excavation under these conditions. Therefore, most of the existing mountain tunnels still choose drilling and blasting method for excavation.

The drilling and blasting method has many advantages, such as simple construction operation, low cost and excellent adaptability, it is these factors make the drilling and blasting method is always favored by the builders and construction parties. However, the uncontrollable nature of the energy in the blasting process often leads to tunnel blasting over-/under-excavation, excessive fragmentation of the surrounding rock, etc., especially in the horizontal soft and hard interlayer blasting conditions, the occurrence frequency of blasting over-/under-excavation and the value of over-/under-break increase explosively, and almost every cycle of blasting will produce different degrees of overbreak and underbreak events^2^. Therefore, in order to improve the poor blasting effect of horizontal stratified surrounding rock, it is necessary to optimize, screen, and pre-evaluate the blasting effect of the horizontal laminated tunnel blasting process.

For the study of blasting effect evaluation, some scholars have carried out evaluation, optimization and prediction research from the aspects of blasting ground vibration^3–6^, blasting flying rock^7^, blasting damage range^8,9^, air overpressure^10^, back break^11^, rock burst^12,13^, rock fragmentation degree after blasting^14–16^, etc.

In addition to the above blasting effects, one of the most important blasting effects for the actual tunnel blasting engineering is the shaping of the contour surface^17^, because the effect of the contour surface will directly determine the difficulty degree of the next cyclic blasting and the efficiency of support implementation. If the blasting forming is poor, some compensatory measures may be taken. Such as replenishment (under-excavation), excessive consumption (over-excavation), irregular surrounding rock contour (improvement or replacement of blasting design scheme), etc. Currently, the research on blasting profile forming effect mainly focuses on the analysis of influencing factors^18,19^, analysis of causal mechanisms^20–22^, and the proposed control measures for profile forming by means of field measurements, equivalent load model calculations or reduced model blasting calculations, physical model tests, optimizers, prediction methods, etc.^23–28^. Among them, in terms of influencing factors, some researches considered blasting design factors (hole spacing, charge diameter, hole layout form, etc.), geological condition factors (burial depth, ground stress conditions, close excavation, water content, temperature, chemical action) as the main inducements leading to over-/under-excavation of actual tunnel engineering blasting^29–32^. Mottahedi et al.^33^ and Mahtab et al.^34^ attributed the causes of over and under excavation phenomena to three categories: controllable factors (blasting conditions), semi-controllable factors (tunnel size, shape), and uncontrollable factors (geological conditions). In terms of causal mechanisms, Lei et al.^35^ suggested that the presence of weak surfaces within the geological body contributed to the reflection of blast stress waves and led to stress redistribution in local areas, which in turn caused an uneven distribution of surrounding rock damage. Ma et al.^36^ studied the propagation characteristics of blasting stress waves and the evolution process of blasting damage in homogeneous and heterogeneous surrounding rocks by means of numerical calculations. The results showed that the superposition of stress waves and the reflection of tensile waves exacerbated the damage degree of surrounding rocks near the bedding. In terms of control measures, measures such as reducing the charge in a single hole, reducing the perimeter hole spacing or adding one to two rows of buffer holes, and simultaneously detonating the explosives in the perimeter holes^37–41^ have been proven to effectively reduce the destructive effect of blasting on rocks outside the excavation contour, and including seismic imaging, vibration monitoring and computer-aided drilling^42,43^ have also been applied to actual tunnel construction projects to effectively control the accuracy of the borehole and the extent of damage to the surrounding rock, with the ultimate aim of improving contour formation. In addition, with the continuous development of methods such as image recognition, machine learning, and deep learning, various fields are very interested in such methods^44,45^, including the engineering field. Especially in recent years, many researchers have tended to use intelligent algorithms to study and evaluate the construction process of blasting projects such as tunnels and roadways^33,42,46,47^, for example, Jang et al.^48^ proposed an empirical method that can evaluate and manage tunnel drilling and blasting overbreak based on several sets of field measurements, which essentially uses an artificial neural network algorithm model to analyze the impact of six geological factors (rock strength, degree of weathering and denudation, structural surface characteristics parameters, etc.) on overbreak. Liu and Liu^49^ used algorithm of hybrid genetic and improved support vector regression to describe the relationship between input (geological conditions, control indicators) and output (smooth blasting parameters) using 18 on-site smooth blasting experimental data as training samples, thus achieving the objective of optimizing tunnel smooth blasting parameters. Koopialipoor et al.^24^ collected 406 sets of on-site tunnel blasting data and used these data as samples to predict tunnel blasting overbreak using algorithms coupled with genetic algorithms and artificial neural networks. Khandelwal^50^ and Saghatforoush et al.^51^ investigated the overbreak problem in underground geotechnical blasting construction using particle swarm optimization and ant colony optimization.

The above-mentioned literatures have analyzed the optimization of tunnel blasting parameters and the assessment of blasting contouring effects from different perspectives, but the blasting optimization study for the special rock formation of the near-horizontal soft and hard interlayer surrounding rocks is not very adequate; secondly, the selection of tunnel blasting solutions is the result of a combination of many factors, indicators and levels, while the previous blasting solutions were determined by a single factor or multiple factors intuitively, which is highly subjective and susceptible to the influence of experience and cannot accurately reflect the actual situation. Based on this, a display dynamic analysis program was used to calculate the blasting simulation results of a layered rock tunnel in southwest China, and compared with the actual engineering blasting results to verify the effectiveness and reliability of the simulation. Subsequently, based on the theoretical achievements of the relevant fuzzy comprehensive evaluation method, a blasting effect evaluation system for tunnels with sand and mud interbedded surrounding rocks was established by comprehensively considering both blasting over excavation and under excavation and tunnel surrounding rock blasting damage. and the blasting effect of the original blasting scheme and the optimized blasting scheme of the sand and mud interlayer tunnel envelope were evaluated using hierarchical analysis to determine the feasibility of the optimized scheme. Finally, the optimized scheme was applied to the tunnel construction site, and the results show that the on-site measured results were in good agreement with the simulation results.

## Quality evaluation criteria for tunnel smooth blasting

Due to the complexity of the influencing factors on the construction site, it is difficult to obtain accurate and reliable blasting parameters only through formula calculation. so it is necessary to screen the blasting parameters through field tests and gradually make the parameters reach optimum values to achieve the desired blasting effect.

The only criterion for judging whether blasting parameters are scientific, reasonable, and effective is the quality of the final blasting profile. Based on this, combined with the Chinese national standard for quality inspection standard JTJ071-98^52^, the quality evaluation criteria for smooth blasting are obtained as shown in Table 1.

**Table 1:** Quality assessment criteria for smooth blasting^53^.

Number	Options	Hard rock	Medium hard rock	Soft rock
1	Surrounding rocks	Unflaking	Unflaking	No major collapses
2	Maximum linear overbreak/cm	< 20	< 25	< 25
3	Local underbreak/cm	< 5	< 5	< 5
4	Explosives Utilization rate/%	≥ 90	≥ 95	100
5	Half-borehole ratio/%	≥ 80	≥ 70	≥ 50
6	Roughness/cm			± 15
7	Vibrational velocity of the mass at a distance of 1 × the diameter of the palm face/cm s^−1^	< 12	< 8	< 5

## Numerical simulation of tunnel blasting

### Project overview

**Figure 1:**
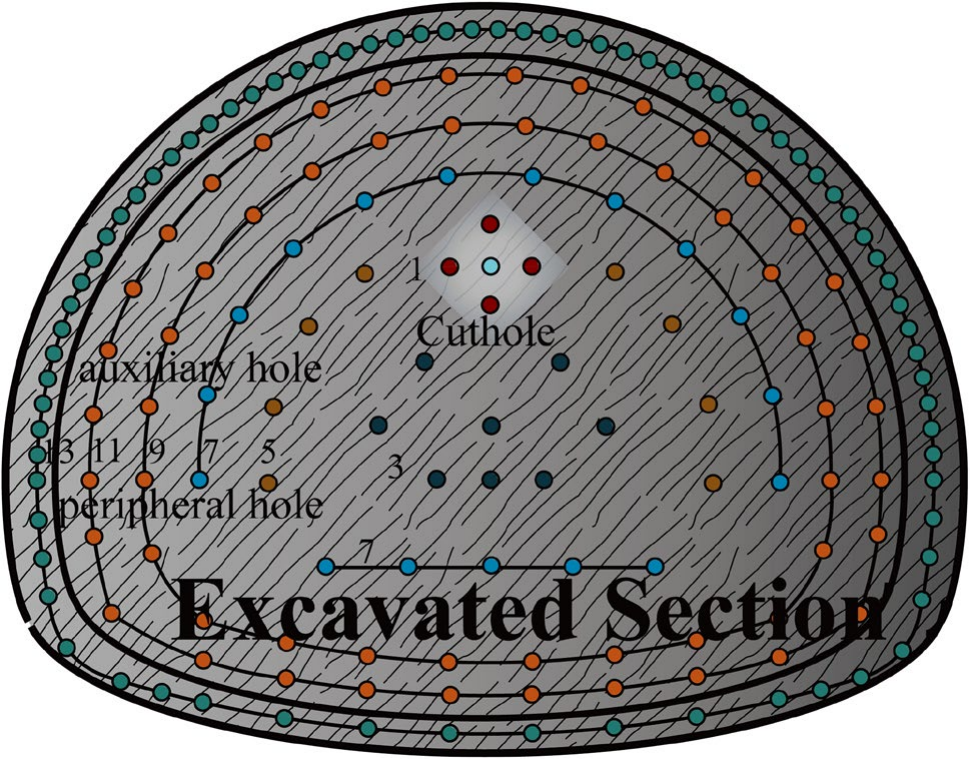
Blast hole distribution.

The overall rock quality is highly weathered with an average degree of interbedded cementation. In addition, the nature of the intersection between the sandstone and mudstone is poor, which needs to be focused on in the subsequent modelling. In view of the special characteristics of the sand-mud interbedded rocks and the fact that construction under such stratigraphic conditions is likely to cause instability such as tunnel collapse, the blasting construction of the sand-mud interbedded section is selected as the object of study in this paper. In the actual project, the explosive charge diameter of 32 mm, perimeter hole spacing of 50 cm, the design footage of 1.2 m, the rest of the blasting parameters are detailed in Table 2, and the layout of blast holes is shown in Fig. 1. The detailed overview of the tunnel project, geological profile, geological lithology, rock mineral composition, and physical and mechanical properties can be found in the Supplementary Material (Supplementary Information 1).

**Table 2:** Tunnel excavation blasting parameter.

Number	Borehole nomenclature	Hole diameter (mm)	Hole depth (m)	Number of holes	Charge structure	Single hole charge (kg)	Subtotal (kg)	Detonation millisecond detonator level
1	cut hole	42	1.5	5	continuation	0.6	3	1
2	Slot hole	42	1.3	8	continuation	0.4	3.2	3
3	auxiliary hole	42	1.3	48	continuation	0.4	19.2	5,7,9,11
4	second stage hole	42	1.3	29	continuation	0.3	8.7	9
5	bottom hole	42	1.3	19	continuation	0.3	5.7	7
6	peripheral hole	42	1.3	55	interval	0.15	8.25	13
7	total			164			48.05

### Finite element modelling

**Figure 2:**
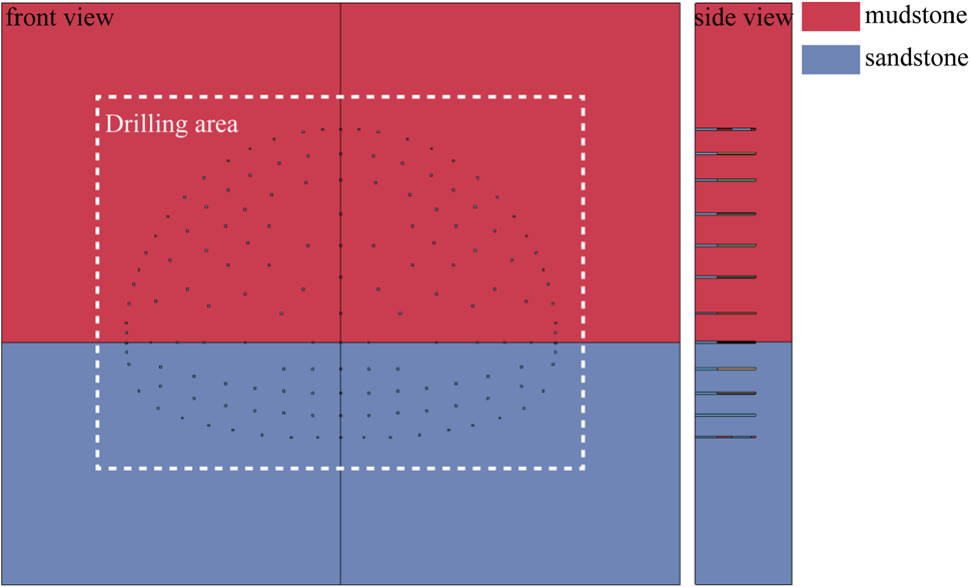
Model schematic.

Based on the geometric profile of the tunnel, a three-dimensional blasting finite element model containing the surrounding rock, explosives and air was developed. After the geometric model was constructed, the model mesh was divided using a variety of meshing methods, followed by setting the bottom region as a fixed displacement boundary condition and the top as a free surface, with reflection-free boundaries added all around the model. In order to fully reflect the mechanical relationship between the sandstone and mudstone, a surface slip contact model was set up between the sandstone and mudstone layers. The established 3D model and the internal structure are shown in Fig. 2, where the red area represents mudstone and the blue area represents sandstone. The validation results of the numerical simulation can be found as Supplementary Material (Supplementary Information 2).

In the process of constructing the model, the meshing of the model is one of the most important aspects. As the model contains explosive bodies, air gaps and rock masses, and the number of gun holes and air gaps involved in the blasting programme is large, the geometry of the whole rock model is extremely irregular. In order to ensure the quality of the cells and the convenience of meshing, a combination of Volumes and Mapped method of meshing is used to partition the rock masses to effectively ensure the overall model cell quality.

### Material parameters

The solid material in the numerical simulation consisted of sandstone and mudstone. Prior to the blasting simulation, the basic mechanical parameters of these two rocks need to be measured to provide input parameters for the subsequent blasting simulation. To this end, uniaxial and triaxial compression tests were carried out on sandstone and mudstone to test and calculate the mechanical parameters such as compressive strength, Poisson's ratio and elastic modulus of the two rocks. The parameters for the rocks, explosives and air are shown in Tables 3, 4, and 5.

**Table 3:** Rock mechanical parameters.

Lithology	Density (Kg/m^3^)	Elastic modulus (GPa)	Poisson's ratio	Uniaxial compressive strength (MPa)	Tensile strength (MPa)	Internal friction angle (°)	Cohesive force (MPa)
Mudstone	2550	1.70	0.29	55.2	2.44	27.35	0.36
Sandstone	2500	8.90	0.19	129.80	6.71	34.77	1.40
interlayer	–	–	–	–	–	35	0.01

**Table 4:** Emulsion explosive parameters.

Material	Density (kg m^−3^)	Detonation velocity (cm μs^−1^)	PCJ burst pressure (10^5^ MPa)	A	B	R1	R2	ω	E_0_
Emulsion explosive	1300	0.400	0.052	326.42	5.80	5.80	1.56	0.57	2.67

**Table 5:** Air parameters.

Material	Density (kg m^−3^)	C_0_	C_1_	C_2_	C_3_	C_4_	C_5_
Air	1.22	0	0	0	0.4	0.4	0

### Design solutions and calculation results

**Figure 3:**
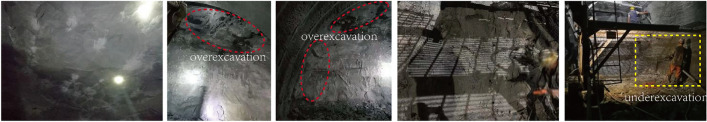
Field blasting effect.

**Figure 4:**
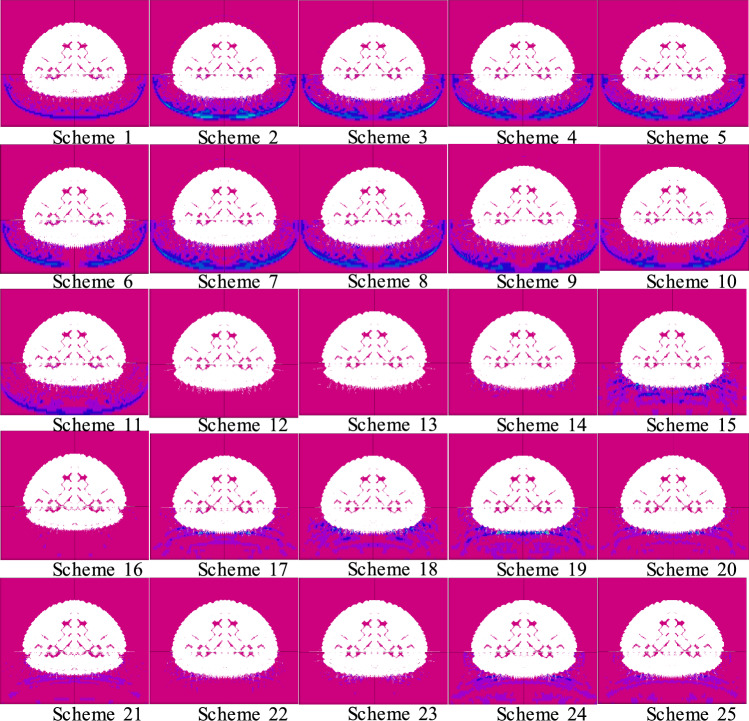
Blasting simulation results of sand-mud interbedded rock.

In view of the urgent need to improve the blasting results of the original scheme at the site (Fig. 3), the blasting plan of sand-mud interbed surrounding rock was developed, a sand-mud interbedded perimeter rock blasting scheme was developed, using a five-factor, five-level orthogonal test with a total of 25 sets of tests, as shown in Table 6. Blasting simulations were carried out for these 25 sets of tests and the results are shown in Fig. 4. The degree of influence of different factors on the tunnel blasting effect can be found as Supplementary Material (Supplementary Information 3).

**Table 6:** Sand-mud surrounding rock blasting design scheme.

Scheme number	1 (millisecond)	2 (uncoupling coefficient)	3 (hole spacing)	4 (charge quantity)	5 (charge concentration)	Maximum linear over-excavation
1	1 (3 ms)	1 (1.44)	1 (32 cm)	1 (43.04 kg)	1 (0.09)	16.8 cm
2	1 (3 ms)	2 (1.52)	2 (34 cm)	2 (43.75 kg)	2 (0.10)	15.3 cm
3	1 (3 ms)	3 (1.6)	3 (36 cm)	3 (44.46 kg)	3 (0.11)	19.8 cm
4	1 (3 ms)	4 (1.68)	4 (38 cm)	4 (44.85 kg)	4 (0.12)	33.6 cm
5	1 (3 ms)	5 (1.76)	5 (40 cm)	5 (45.89 kg)	5 (0.13)	6.5 cm
6	2 (5 ms)	1 (1.44)	2 (34 cm)	3 (44.46 kg)	4 (0.12)	33.6 cm
7	2 (5 ms)	2 (1.52)	3 (36 cm)	4 (44.85 kg)	5 (0.13)	17.0 cm
8	2 (5 ms)	3 (1.6)	4 (38 cm)	5 (45.89 kg)	1 (0.09)	15.6 cm
9	2 (5 ms)	4 (1.68)	5 (40 cm)	1 (43.04 kg)	2 (0.10)	7.8 cm
10	2 (5 ms)	5 (1.76)	1 (32 cm)	2 (43.75 kg)	3 (0.11)	18.2 cm
11	3 (7 ms)	1 (1.44)	3 (36 cm)	5 (45.89 kg)	2 (0.10)	25.2 cm
12	3 (7 ms)	2 (1.52)	4 (38 cm)	1 (43.04 kg)	3 (0.11)	20.2 cm
13	3 (7 ms)	3 (1.6)	5 (40 cm)	2 (43.75 kg)	4 (0.12)	25.2 cm
14	3 (7 ms)	4 (1.68)	1 (32 cm)	3 (44.46 kg)	5 (0.13)	25.2 cm
15	3 (7 ms)	5 (1.76)	2 (34 cm)	4 (44.85 kg)	1 (0.09)	16.8 cm
16	4 (10 ms)	1 (1.44)	4 (38 cm)	2 (43.75 kg)	5 (0.13)	16.6 cm
17	4 (10 ms)	2 (1.52)	5 (40 cm)	3 (44.46 kg)	1 (0.09)	20.1 cm
18	4 (10 ms)	3 (1.6)	1 (32 cm)	4 (44.85 kg)	2 (0.10)	21.3 cm
19	4 (10 ms)	4 (1.68)	2 (34 cm)	5 (45.89 kg)	3 (0.11)	25.2 cm
20	4 (10 ms)	5 (1.76)	3 (36 cm)	1 (43.04 kg)	4 (0.12)	25.2 cm
21	5 (12 ms)	1 (1.44)	5 (40 cm)	4 (44.85 kg)	3 (0.11)	16.8 cm
22	5 (12 ms)	2 (1.52)	1 (32 cm)	5 (45.89 kg)	4 (0.12)	31.1 cm
23	5 (12 ms)	3 (1.6)	2 (34 cm)	1 (43.04 kg)	5 (0.13)	18.5 cm
24	5 (12 ms)	4 (1.68)	3 (36 cm)	2 (43.75 kg)	1 (0.09)	21.8 cm
25	5 (12 ms)	5 (1.76)	4 (38 cm)	3 (44.46 kg)	2 (0.10)	16.8 cm

Comparing the simulation results of each scheme, Scheme 6 and Scheme 10 were preliminarily selected. However, considering the on-site testing and testing costs of Scheme 6 and Scheme 10, further evaluation of these two schemes is still needed in this article.

## Comprehensive evaluation of the blasting programme

### Safety evaluation system and indicators

**Figure 5:**
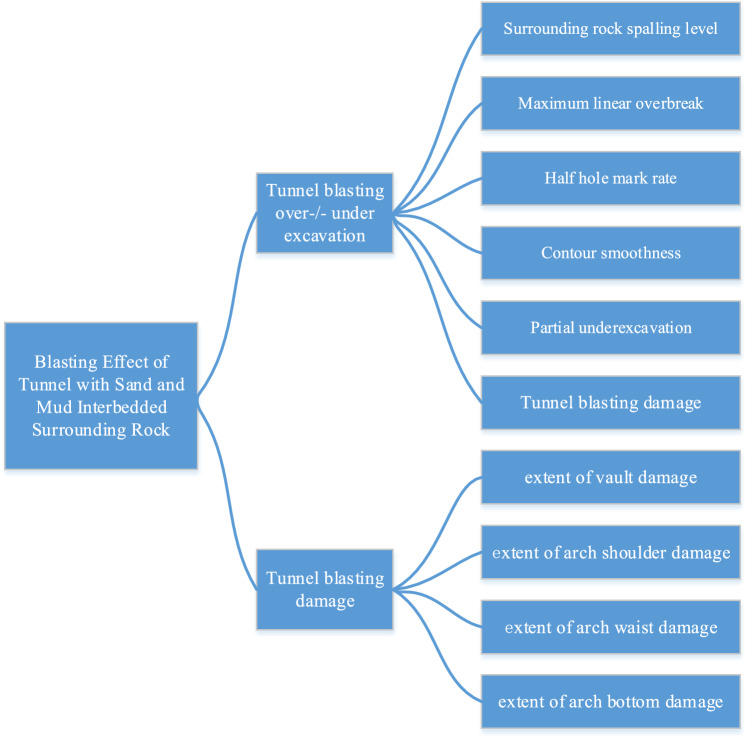
Blasting effect evaluation system of sand-mud interbedded surrounding rock tunnel.

In order to accurately assess the results of the proposed solution, a multi-layer, multi-item comprehensive evaluation system was further developed with the aid of fuzzy theory and hierarchical analysis based on the site construction conditions. In this system, the top layer represents the blasting results of interbedded surrounding rock, the middle layer describes the blasting effect of stratified rock, and the bottom layer represents the blasting effect index of stratified rock tunnel. The constructed blasting effect evaluation index and evaluation system of sand-mud interbedded surrounding rock tunnel are shown in Fig. 5.

The hierarchical factor set can be obtained as follows:

1. First level factor set.
  U = {U_1_, U_2_} = {tunnel blasting over-/under-excavation, tunnel blasting damage}.
2. Second level factor set.
  U_1_ = {u_11_, u_12_, u_13_, u_14_, u_15_} = {degree of surrounding rock spalling, maximum linear overbreak, local under-excavation amount, half-hole mark rate, contour flatness}, U_2_ = {u_21_, u_22_, u_23_, u_24_} = {extent of vault damage, extent of arch shoulder damage, extent of arch waist damage, extent of arch bottom damage}.

### Comment set

Combined with the relevant industry blasting effect level classification, the blasting effect of sand-mud interbedded surrounding rock tunnels is divided into 5 levels, Level I is very good, Level II is good, Level III is general, Level IV is relatively poor, and Level V is poor. In order to quantitatively analyze the effect of tunnel blasting, a percentage system was used to express the stability situation, as shown in Table 7. In order to quantitatively analyze the effect of tunnel blasting, a percentage system was used to express the stability situation, as shown in Table 5.

**Table 7:** Blasting effect level of sand and mud interbedded surrounding rock tunnel.

Effect level	Level I	Level II	Level III	Level IV	Level V
Score interval	[90, 100]	[80, 90)	[70, 80)	[60, 70)	[0, 60)

The final set of decision comments is V = {v1, v2, v3, v4, v5}, corresponding to 5 levels of very good, good, general, relatively poor, and poor.

### Calculation of evaluation index weight

The evaluation index system established above is analysed and a judgement matrix is established. Subsequently, the maximum eigenvalues and maximum eigenvectors after normalization are calculated, and the corresponding weights of each factor are obtained using the eig command in Matlab. The matrix constructed this time and the results are as shown in Tables 8, 9 and 10.

**Table 8:** First-level factor set U judgment matrix, consistency check and weight value.

	U_1_	U_2_	W_i_	Consistency check
U_1_	1	2	0.6667	*λ*_max_ $={\sum }_{i=1}^{n}\frac{{\left(AW\right)}_{I}}{n{W}_{i}}$ *CI* = (*λ*_max_ − *n*)/(*n* − 1) *CR* = *CI*/*RI*
U_2_	0.5	1	0.3333

**Table 9:** Second-level factor set U_1_ judgment matrix, consistency check and weight value.

	u_11_	u_12_	u_13_	u_14_	u_15_	W_1i_	Consistency check
u_11_	1	1/4	1/4	1	1/2	0.0813	*λ*_max=_${\sum }_{i=1}^{n}\frac{{\left(AW\right)}_{I}}{n{W}_{i}}$ *CI* = (*λ*_max_ − *n*)/(*n* − 1) *CR* = *CI*/*RI*
u_12_	4	1	1	4	2	0.3253	
u_13_	4	1	1	6	2	0.3553	
u_14_	1	1/4	1/6	1	1/2	0.0754	
u_15_	2	1/2	1/2	2	1	0.1626

**Table 10:** Second-level factor set U_2_ judgment matrix, consistency check and weight value.

	u_21_	u_22_	u_23_	u_24_	W_2i_	Consistency check
u_21_	1	2	2	2	0.3952	*λ*_max_ $={\sum }_{i=1}^{n}\frac{{\left(AW\right)}_{I}}{n{W}_{i}}$ *CI* = (*λ*_max_ − *n*)/(*n* − 1) *CR* = *CI*/*RI*
u_22_	1/2	1	1	2	0.2322	
u_23_	1/2	1	1	2	0.2322	
u_24_	1/2	1/2	1/2	1	0.1404	

### Tunnel blasting over-/under-excavation scoring model

For the first factor set of the second layer the surrounding rock itself and the environmental parameters U_1_ = {u_11_, u_12_, u_13_, u_14_, u_15_} = {degree of surrounding rock spalling, maximum linear overbreak, local under-excavation amount, half-hole mark rate, contour flatness}, are analyzed separately. Afterwards, referring to scoring models for similar engineering evaluations, the final evaluation model for blasting over-/under-excavation in the blasting effect system of sand-mud interbedded surrounding rock tunnels is shown in Table 11.

**Table 11:** Scoring model for blasting over-/under-break of tunnel in interbedded surrounding rock.

Index	Score calculation method			
Level of surrounding rock spalling	Severe spalling	Spalling	Slight spalling	No spalling
Score	60	(70, 80)	(80, 90)	(90, 100)
Maximum linear overbreak	> 20 cm	10–20 cm	5–10 cm	< 5 cm
Score	60	(70, 80)	(80, 90)	> 90分
Local under-excavation amount	> 20 cm	10–20 cm	5–10 cm	< 5 cm
Score	60	(70, 80)	(80, 90)	> 90分
Half-hole mark rate	< 70%	70–80%	80–90%	> 90%
Score	< 60	(60, 70)	(70, 80)	> 90
Contour flatness	Relatively poor	General	Relatively flat	Flat
Score	70	80	90	100

For the second factor set of the second layer U_2_ = {u_21_, u_22_, u_23_, u_24_} = {vault damage, arch shoulder damage, arch waist damage, arch bottom damage}, the final blast damage scoring model is shown in Table 12.

**Table 12:** Tunnel blasting damage scoring model.

Index	Score calculation method			
Vault damage	No damage	Lower degree of damage	Relatively higher degree of damage	High degree of damage
Score	(90, 100)	(80, 90)	(70, 80)	60
Arch shoulder damage	No damage	Lower degree of damage	Relatively higher degree of damage	High degree of damage
Score	(90, 100)	(80, 90)	(70, 80)	60
Arch waist damage	No damage	Lower degree of damage	Relatively higher degree of damage	High degree of damage
Score	(90, 100)	(80, 90)	(70, 80)	60
Arch bottom damage	No damage	Lower degree of damage	Relatively higher degree of damage	High degree of damage
Score	(90, 100)	(80, 90)	(70, 80)	60

### Assessment results

#### Comprehensive evaluation results of the original design blasting scheme of the project

Based on the blasting simulation results of the original tunnel scheme, the evaluation model and secondary indicators were evaluated according to the above scoring model, and the scores of each secondary indicator were obtained as shown in Table 13.

**Table 13:** Original design secondary indicators and scores.

Indicators	Sub-indicators	Specific situation	Score
Blasting over-/under-excavation	Degree of surrounding rock spalling	Severe spalling	70
	Maximum linear overbreak	No overbreak	90
	Local under-excavation amount	> 20 cm	60
	Half-hole mark rate	< 70%	60
	Contour flatness	Poor flatness	70
Blasting damage	Vault damage	No damage	95
	Arch shoulder damage	No damage	95
	Arch waist damage	Relatively higher degree of damage	75
	Arch bottom damage	Relatively higher degree of damage	75

The score of each indicator is calculated according to the scoring model of each indicator, which is substituted into the following trapezoidal distribution affiliation function and normalized to obtain the fuzzy synthesis matrix *R*. In view of the fact that the larger the scores of the evaluation indicators in Table 13, the better the corresponding blasting effect, the biased large trapezoidal distribution affiliation function is used.

1 $$f_{1} \left( {\mu_{ij} } \right) = \left\{ \begin{gathered} 1\left( {90 \le \mu_{ij} \le 100} \right) \hfill \\ \frac{{\mu_{ij} - 80}}{90 - 80}\left( {80 \le \mu_{ij} < 90} \right) \hfill \\ 0\left( {\mu_{ij} < 80} \right) \hfill \\ \end{gathered} \right.$$

2 $$f_{2} \left( {\mu_{ij} } \right) = \left\{ \begin{gathered} \frac{{100 - \mu_{ij} }}{100 - 90}\left( {90 \le \mu_{ij} \le 100} \right) \hfill \\ 1\left( {80 \le \mu_{ij} < 90} \right) \hfill \\ \frac{{\mu_{ij} - 70}}{80 - 70}\left( {70 \le \mu_{ij} < 80} \right) \hfill \\ 0\left( {\mu_{ij} < 70} \right) \hfill \\ \end{gathered} \right.$$

3 $$f_{3} \left( {\mu_{ij} } \right) = \left\{ \begin{gathered} 0\left( {90 \le \mu_{ij} \le 100} \right) \hfill \\ \frac{{90 - \mu_{ij} }}{90 - 80}\left( {80 \le \mu_{ij} < 90} \right) \hfill \\ 1\left( {70 \le \mu_{ij} < 80} \right) \hfill \\ \frac{{\mu_{ij} - 60}}{70 - 60}\left( {60 \le \mu_{ij} < 70} \right) \hfill \\ 0\left( {\mu_{ij} < 60} \right) \hfill \\ \end{gathered} \right.$$

4 $$f_{4} \left( {\mu_{ij} } \right) = \left\{ \begin{gathered} 0\left( {80 \le \mu_{ij} \le 100} \right) \hfill \\ \frac{{80 - \mu_{ij} }}{80 - 70}\left( {70 \le \mu_{ij} < 80} \right) \hfill \\ 1\left( {60 \le \mu_{ij} < 70} \right) \hfill \\ \frac{{\mu_{ij} - 50}}{60 - 50}\left( {50 \le \mu_{ij} < 60} \right) \hfill \\ 0\left( {\mu_{ij} < 50} \right) \hfill \\ \end{gathered} \right.$$

5 $$f_{5} \left( {\mu_{ij} } \right) = \left\{ \begin{gathered} 0\left( {70 \le \mu_{ij} \le 100} \right) \hfill \\ \frac{{70 - \mu_{ij} }}{70 - 60}\left( {60 \le \mu_{ij} < 70} \right) \hfill \\ 1\left( {0 \le \mu_{ij} < 60} \right) \hfill \\ \end{gathered} \right.$$

The results of the single-factor evaluation are obtained by multiplying *B* = W*R with the corresponding weight vectors respectively.

Based on the primary assessment indicator weights W = [0.67 0.33] derived above using hierarchical analysis, the results of the single factor assessments were combined to form a total secondary assessment matrix *R* as.

6 $$R = \left[ \begin{gathered} B_{1} \hfill \\ B_{2} \hfill \\ B_{3} \hfill \\ \end{gathered} \right]$$

According to the two-level fuzzy comprehensive evaluation model, the overall comprehensive evaluation of the system can be carried out, and the corresponding evaluation results can be obtained in accordance with the principle of maximum affiliation.

7 $$B = W * R$$

The total assessment score for this system was calculated as *P* = B*v^T^ = 77.3145 based on the mean of the effect level scores, which indicates that the original blasting effect level of the sand and mud interlayer tunnel was Class III, i.e. the blasting effect is general.

#### Fuzzy composite assessment results of the optimized blasting solution

Based on the results of the blasting optimization, the various indicators in the tunnel blasting effect evaluation system are re-scored as shown in Table 14.

**Table 14:** Optimized profile and scores for each secondary indicator.

Indicators	Sub-indicators	Specific situation	Score
Blasting over-/under-excavation	Degree of surrounding rock spalling	No spalling	95
	Maximum linear overbreak	< 5 cm	90
	Local under-excavation amount	< 5 cm	90
	Half-hole mark rate	80%	70
	Contour flatness	Flat	100
Blasting damage	Vault damage	No damage	95
	Arch shoulder damage	No damage	95
	Arch waist damage	No damage	95
	Arch bottom damage	Lower degree of damage	85

According to the principle of maximum membership, the corresponding evaluation results are.

8 $$B{\prime} = W * R{\prime}$$

The total assessment score for this system was calculated from Table 7 as *P* = B*v^T^ = 91.2765, indicating an optimized tunnel blasting effectiveness rating of Class I i.e. very good blasting effectiveness.

#### Analysis of field test results

**Figure 6:**
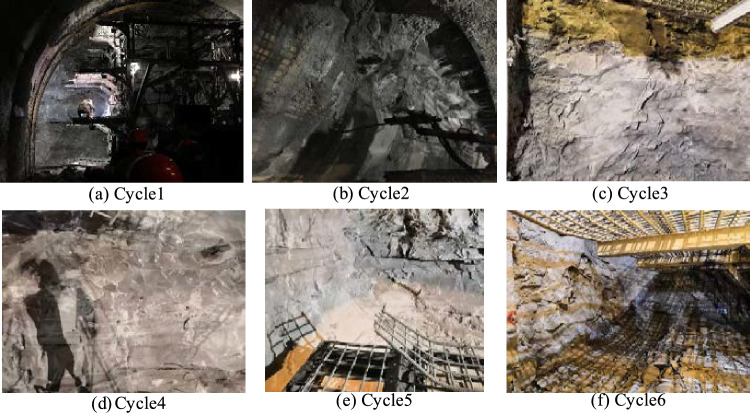
Optimized site blasting effect.

**Figure 7:**
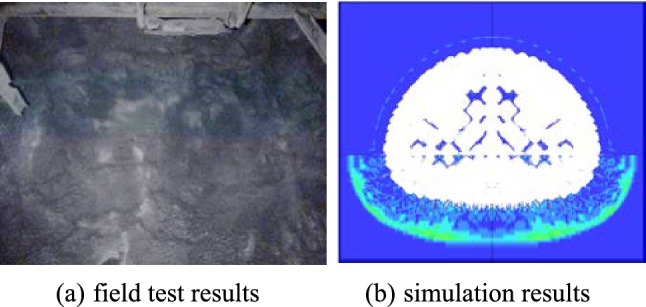
Comparison of results. (**a**) field test results, (**b**) simulation results.

When adjusting section blasting solutions, the site operator generally relies on the existing surrounding rock grading system and rarely takes into account the contact relationship between soft and hard rock, resulting in poor blasting results on site, which suggests that there is a lot of uncertainty in relying on practical experience alone to obtain a better blasting solution. In this paper, numerical simulations were carried out to take into account this variation in the mechanical properties of the surrounding rocks, and were able to reflect the blasting damage characteristics of the laminated surrounding rocks, which suggests that the blasting simulations were more appropriate to the actual situation. The proposed solution was then evaluated using a comprehensive assessment method and the corresponding results show that the proposed solution can improve the blasting results. Finally, after communication and discussion with the site manager and the construction team regarding the implementation of the specific solution, the construction process and the charging process, the blasting solution was applied to the cyclic blasting on site and the blasting results applied (Fig. 6) show that the use of the optimized solution can improve the flatness of the tunnel section and can significantly reduce the amount of over-/under-excavation. In addition, a comparison of the test results with the simulated results showed that the linear over-/under-excavation in the field test was generally consistent with the simulated values (Fig. 7 and Table 15), thus demonstrating the validity of the blasting simulation and the feasibility of the proposed solution.

**Table 15:** Comparison of simulated and measured values after optimization with sand-mud interbedded rock.

Sand-mud interbedded rock	Linear overbreak of arch crown	Excavation amount	Surrounding rock	Contour flatness
Measured value	< 3.5 cm	< 4.5 cm	No spalling	Relatively flat
Simulated value	< 3 cm	≤ 5 cm	No spalling	Relatively flat

## Conclusion

The main conclusions obtained in this paper are as follows:

1. Based on the actual tunnel stratigraphic conditions and the site blasting solution, the blasting process of the laminated rock tunnel was calculated using finite element calculations, which showed that there was over-/under-excavation in the junction area between the sandstone and mudstone of the tunnel under the original solution conditions, with the under-excavation range reaching 35 cm. This calculation result is very close to the actual situation, which indicates the reliability of the numerical simulation.
2. Based on the simulations, a five-factor, five-level orthogonal test scheme was designed for the detonation interval (3–12 ms), perimeter hole spacing (32–40 cm), uncoupling factor (1.44–1.76), charge volume (43.04–45.89 kg) and charge concentration (0.09–0.13).
3. The blasting effect of the original scheme and the optimized scheme was evaluated. The results showed that the total score of the original scheme was only 77.3145, while the total score of the optimized scheme is 91.2765.
4. The optimized scheme was applied to a tunnel project in southwest China, and the final test results showed that the optimized scheme can better control the forming flatness of the tunnel contour and the phenomenon of over-/under-excavation, with the maximum over-excavation within 50 mm, thus demonstrating the effectiveness of the selected optimised solution and the feasibility of the hybrid simulation-assessment evaluation method (Supplementary Information).

### Supplementary Information

Supplementary Information.

## Data Availability

All data used during the study appear in the submitted article.
