# Supplementary material for: Blasting profile evaluation of sand-mud interbedded surrounding rock during the large-span tunnel construction
Source: Sci Rep. 2024 May 30;14:12405. doi: 10.1038/s41598-024-62921-3 (PMC11136968; doi:10.1038/s41598-024-62921-3)
Supplement: Supplementary file 1 — Supplementary Information. [file 41598_2024_62921_MOESM1_ESM.pdf]

Blasting profile evaluation of sand-mud interbedded surrounding rock during the large-span tunnel construction

LongHao Ma [1], Fei Lin [2,3,4], Yanqiang Du [†,1], Song Ren [†,2,4], NengZeng Long [2,4], Ping Zhang [2,4]

([1] *School of Civil Engineering, Luoyang Institute of Science and Technology, Henan 471023 Henan, China*)

([2] *School of Resources and Safety Engineering, Chongqing University, Chongqing 400030 Chongqing, China*)

([3] *China Coal Technology Engineering Group Huaibei Blasting Technology Research Institute Limited Company, Huaibei, Anhui 235099, P.R. China*)

([4] *State Key Laboratory for the Coal Mine Disaster Dynamics and Controls, Chongiqng University, 400044 Chongqing, China*)

Supplementary information 1

The Simianshan Tunnel is located in Berlin Town, Jiangjin District, Chongqing, China, about 10km away from the Simianshan Scenic Spot and about 60km away from the urban area of Jiangjin. The tunnel, as a key construction project in Southwest China, has a length of 4,880 m and 4,875 m for the left and right lines, respectively, with the maximum permissible longitudinal slope of the tunnel is 3% and the maximum depth of burial is 615 m, and the tunnel cross-sectional structure is consistent with most current tunnel cross-sectional profiles, which are circular curved wall structures. The Simianshan Tunnel is a two-lane tunnel, with the design speed of 80 km. The design spacing between the left and right lines of the tunnel is 22 m as the demarcation point of the small-clearance tunnel and the separated tunnel, and the design line spacing of 15.4-60.8 m is adopted as the structural form of the combined small-clearance and separated tunnel. The longitudinal geology along the tunnel axis is shown in Supplementary Figure S1.

The main lithology of the tunnel site is mudstone, sandstone and sand-mud interbedded rock, and the entrance and exit section is partly located in silty clay interbedded stone. Among them, the sandstone is grayish-white with medium-fine grained structure, and the main mineral components are clay minerals such as kaolin and chlorite and quartz (quartz accounts for 70%-80%, clay minerals account for 20%-30%). The mudstone is purplish red and has a argillaceous structure. Its mineral composition is 60%-70% quartz and 30%-40% clay minerals. The natural water content of mudstone is 0.491%. Detailed mechanical parameters of sandstones and mudstones can be obtained from basic rock mechanical tests, which will be expanded in the Material Parameters section.

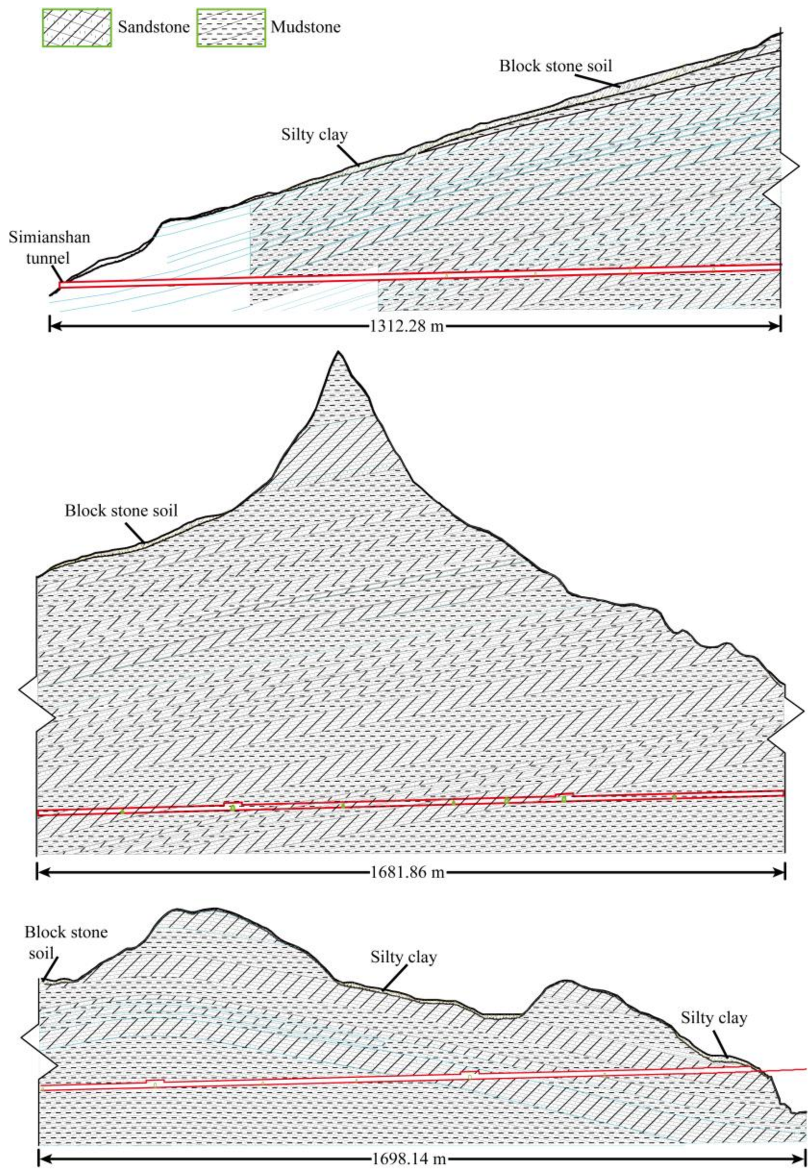

Supplementary Figure S1. Geological profile for the right line of the Simianshan tunnel

Supplementary information 2

In order to verify the effectiveness of the simulation, the differences between numerical simulation and experimental fracture modes were compared based on existing experimental research.[54] The experimental model consists of a concrete block measuring 3 meters in length, 3 meters in width, and 1.5 meters in height, with a 38 mm diameter blast hole set at the center of the block. The parameters of explosives and concrete in the model are completely consistent with existing research. The calculated form of concrete blasting failure is shown in Supplementary Figure S2. Compared with existing research, the simulated failure form, crack number, and crack bifurcation form are highly consistent with experimental results, indicating that the numerical simulation results are reliable.

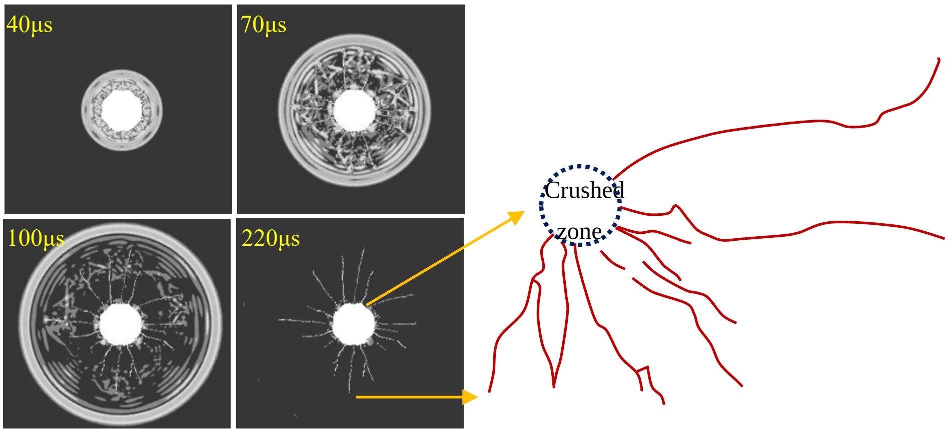

(a)Simulation result (b)Experimental result

Supplementary Figure S2. Result comparison

Supplementary information 3

Analysis of blasting influencing factors

In order to further analyze the degree of influence of various factors on the blasting effect, the range analysis method is used to explore the range value R_j and sensitivity level of each influencing factor. R_j is calculated according to the Supplementary Equation S1.[55]

$$K_{ij} = \frac{1}{n_{ij}} \sum_{k=1}^{n_{ij}} Y_k$$
$$R_j = \max\left\{K_{1j}, K_{2j}, \cdots\right\} - \min\left\{K_{1j}, K_{2j}, \cdots\right\} \tag{S1}$$

Where K_{ij} represents the mean of all test results for factor j at level i. n_{ij} is the number of trials for factor j at level i. Y_k is the blasting result of the kth test, k=1, 2,$\cdots n_{ij}$.

The range analysis results of each influencing factor are shown in Supplementary Table S1. It can be seen that the sensitivity of each factor is ranked as: 5-3-2-4-1, in which the concentration of charge has the highest degree of influence on the maximum linear over-excavation of the tunnel peripheral surrounding rock, followed by the hole spacing, the uncoupling coefficient and the amount of charge, and the influence of the micro-difference is weaker than the other factors.

Factor	1(Millisecond)	2(Uncoupling coefficient)	3(Hole spacing)	4(Charge quantity)	5(Charge concentration)
K_1	18.4	21.8	22.5	17.7	18.2
K_2	18.4	20.7	21.9	19.4	17.3
K_3	22.5	20.1	21.8	23.1	20.0
K_4	21.7	22.7	20.6	21.1	29.7
K_5	21.0	16.7	15.3	20.7	16.8
R_j	4.1	6.0	7.2	5.4	12.9
rank	5	3	2	4	1

Supplementary Table S1. Range analysis of each factor

References

54. Onederra, I., Furtney, J., Sellers, E. & Iverson, S. Modelling blast induced damage from a fully coupled explosive charge. *Int J Rock Mech Min Sci* **58,** 73–84, doi:10.1016/j.ijrmms.2012.10.004 (2013).
55. Guo, S., Zhou, X., Song, SS, Mei, YG & Fang YT. Optimization of leaching conditions for removing sodium from sodium-rich coals by orthogonal experiments. *Fuel* **208,** 499–507, doi: 10.1016/j.fuel.2017.07.032 (2017).
